# Cardiovascular disease risk factor prevalence and estimated 10-year cardiovascular risk scores in Indonesia: The SMARThealth Extend study

**DOI:** 10.1371/journal.pone.0215219

**Published:** 2019-04-30

**Authors:** Asri Maharani, Sujarwoto, Devarsetty Praveen, Delvac Oceandy, Gindo Tampubolon, Anushka Patel

**Affiliations:** 1 Division of Neuroscience and Experimental Psychology, University of Manchester, Manchester, United Kingdom; 2 University of Brawijaya, Malang, Indonesia; 3 The George Institute for Global Health, University of New South Wales, Hyderabad, India; 4 Division of Cardiovascular Science, The University of Manchester, Manchester Academic Health Science Centre, Manchester, United Kingdom; 5 Department of Biomedicine, Faculty of Medicine, Universitas Airlangga, Surabaya, Indonesia; 6 Manchester Institute for Collaborative Research on Aging, University of Manchester, Manchester, United Kingdom; 7 The George Institute for Global Health, University of New South Wales, Sydney, Australia; Makerere University School of Public Health, UGANDA

## Abstract

**Background:**

The brunt of cardiovascular disease (CVD) burden globally now resides within low- and middle-income countries, including Indonesia. However, little is known regarding cardiovascular health in Indonesia. This study aimed to estimate the prevalence of elevated CVD risk in a specific region of Indonesia.

**Methods:**

We conducted full household screening for cardiovascular risk factors among adults aged 40 years and older in 8 villages in Malang District, East Java Province, Indonesia, in 2016–2017. 10-year cardiovascular risk scores were calculated based on the World Health Organization/International Society of Hypertension’s region-specific charts that use age, sex, blood pressure, diabetes status and smoking behaviour.

**Results:**

Among 22,093 participants, 6,455 (29.2%) had high cardiovascular risk, defined as the presence of coronary heart disease, stroke or other atherosclerotic disease; estimated 10-year CVD risk of ≥ 30%; or estimated 10-year CVD risk between 10% to 29% combined with a systolic blood pressure of > 140 mmHg. The prevalence of high CVD risk was greater in urban (31.6%, CI 30.7–32.5%) than in semi-urban (28.7%, CI 27.3–30.1%) and rural areas (26.2%, CI 25.2–27.2%). Only 11% and 1% of all the respondents with high CVD risk were on blood pressure lowering and statins treatment, respectively.

**Conclusions:**

High cardiovascular risk is common among Indonesian adults aged ≥40 years, and rates of preventive treatment are low. Population-based and clinical approaches to preventing CVD should be a priority in both urban and rural areas.

## Introduction

The ongoing demographic transition, combined with epidemiological and nutritional transitions, is contributing to the continued shift of the cardiovascular diseases (CVDs) burden from developed to developing countries [1, 2]. Ischaemic Heart Disease and stroke are the leading cause of death in the middle-income countries and Disability-Adjusted Life Years (DALYs) in most countries [3]. In Indonesia, CVDs are the leading cause of both morbidity and mortality, responsible for a third of all deaths in Indonesia [4]. Years of life lost due to the premature mortality from coronary heart disease (CHD) and cerebrovascular diseases are estimated to be 3,299 and 2,555 years of life lost/100,000, respectively [5].

Prior studies have shown that the high burden of CHD and stroke in the Indonesian population is attributable to preventable vascular risk factors, particularly hypertension, obesity, dyslipidaemia and active tobacco use [6, 7]. Data from the 2008 Indonesian Family Life Survey (IFLS4), found that less than one-third of Indonesians with moderate to high cardiovascular risk were not receiving appropriate treatment [8]. Individuals with higher per capita expenditure have a higher probability to meet their cardiovascular care needs. Marked geographical disparities were revealed, with rural residents being much less likely to receive CVD care. The absence of a universal healthcare insurance scheme is one possible explanation for the demonstrated inequality in cardiovascular care.

In response to inadequate access to health services, the Government of Indonesia launched a national health insurance scheme, the *Jaminan Kesehatan Nasional* (JKN), in 2014 [9, 10]. It aims to contribute towards achieving universal health coverage by enrolling Indonesia’s entire population (275.5 million people) in the JKN by 2019. In the first step, JKN unified insurance programs targeted the poor and near poor, civil servants, army, and pensioners. Since 1 January 2014, JKN membership has been opened to others, where independent members pay personal premiums. By August 2018, 200 million people were already covered [11]. In addition to financial protection, the Government of Indonesia has increased the number of primary health centres, as well as inpatient beds in both public and private hospitals, nationally. Focusing on non-communicable diseases, the Ministry of Health organizes health promotion activities through a community engagement programme called *Pos Pelayanan Terpadu* (*Posbindu*) [12]. *Posbindu* focuses on raising public awareness, early screening and early detection of these conditions. However, despite these measures, there is inadequate information about the cardiovascular health in Indonesia for further planning of service response.

The primary objective of this study was to determine the distribution of CVD risk factors and CVD risk across communities with different level of urbanisation, and subgroups representing a range of sociodemographic characteristics.

## Subjects and methods

The study received ethics approval from the Ethical Committee, Ministry of Research, Technology, and Higher Education, Medical Faculty of Brawijaya University (330/EC/KEPK/08/2016) and was registered on the Clinical Trial Registry of India (CTRI/2017/08/009387). Written informed consent was obtained from all participants contributing data to the analyses.

### Study population and setting

The SMARThealth Extend study is the implementation of a digital health enabled primary care intervention in Malang District, East Java Province, Indonesia. A description of the SMARThealth intervention is available elsewhere [13, 14]. The current study was conducted among adults 40 years of age and older in urban, semi-urban and rural villages of the district of Malang. The Indonesia’s Population Census of 2000 and 2010 classified a village as urban area if all the following criteria are met: i) a population density of at least 5,000 persons/km2; ii) 25% or less of the households works in the agricultural sector; and iii) ten or more specific urban facilities (e.g. schools and hospitals) exist [15]. Rural villages are those where the largest sector is agriculture, and where fewer than 10 specific urban facilities exist. The category of ‘semi-urban’ to was added capture of the reality of Indonesia’s urbanization, and includes areas with population density less than urban areas, but where the majority of the population work in non-agricultural sectors.

Malang is the second largest district in the province of East Java. Its area covers 3,535 square kilometres, with an agricultural emphasis on rice and sugar cane. Malang’s total population is 2,544,315 distributed across 33 sub-districts, 390 villages and 3,125 community neighbourhoods. Malang was selected for this study because its villages represent an urban, semi-urban and rural distribution seen in many parts of Indonesia.

**Fig 1:**
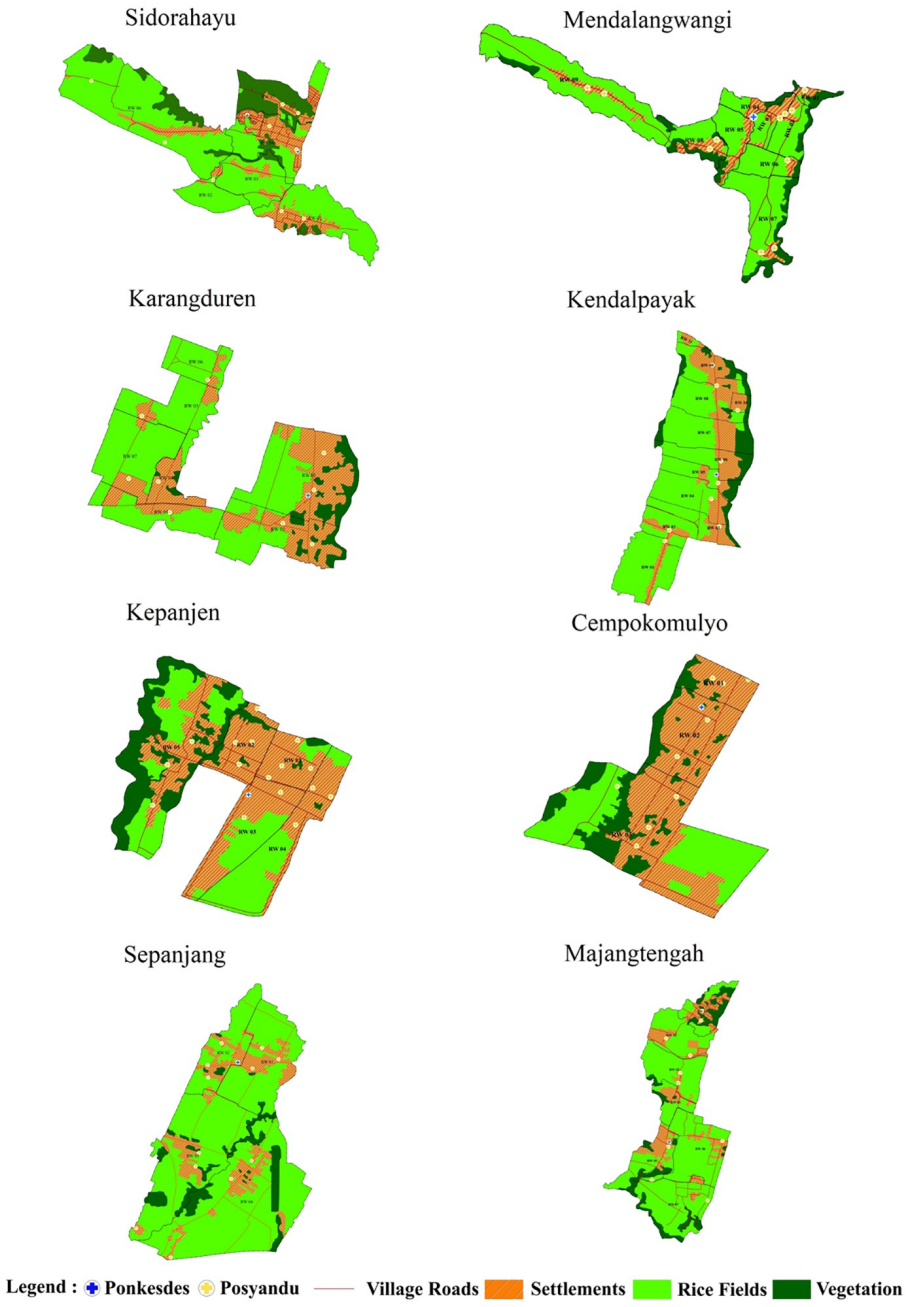
SMARThealth intervention and control villages.

We selected eight villages (Karangduren, Kendalpayak, Kepanjen, Cepokomulyo, Sidorahayu, Mendalanwangi, Sepanjang, and Majangtengah) that representing a range of rurality, occupation of the majority of residents, proximity to a tobacco factory, population density, and number of Kaders (voluntary health workers) (Fig 1, S1 Table). We categorized each village as urban (Karangduren, Kendalpayak, Kepanjen, and Cepokomulyo), semi-urban (Sidorahayu and Mendalanwangi), or rural (Sepanjang and Majangtengah). All adults aged 40 years or older in the eight villages were included in this study.

### Study design

This study was a community-based cross-sectional study in which the data were collected by means of interviews and the taking of physical measurements. No sampling was involved as all eligible residents were invited to participate through complete household visits. The data were collected from January 2016 to March 2016.

### Data collection

The methods for completing the epidemiological questionnaires and for anthropometric measurements were based on a standard protocol [13, 14]. Interviews were performed by trained enumerators who used a questionnaire to collect information on demographic status, socioeconomic status, medical history, family medical history, smoking status, and physical activity levels. The anthropometric measurements included height, weight, and body mass index (BMI). Height measurements were accurate to 1 cm and weight measurements to 1 kg. BMI was calculated as body weight (kg) divided by height squared (m2).

Blood pressure and heart rate were measured using a digital sphygmomanometer (OMRON HEM-7130 made in Japan) with a cuff of appropriate size following the standard recommended procedures. Three blood pressure readings were taken three times at 5-minute intervals after 5 minutes of rest in the sitting position with positioning of the arm, with the average used for data analysis. Random blood glucose rates were measured using the pin-prick method and portable glucometers (FreeStyle Optium Neo) [16].

### Cardiovascular risk factors and 10-year risk for CVD

Participants were categorized as having hypertension if he or she had a history of hypertension diagnosed by a physician, was on hypertension treatment, or had a systolic blood pressure (SBP) ≥140 mmHg and/or a diastolic blood pressure ≥90 mmHg. A participant was considered to have diabetes mellitus if he or she had a previous physician diagnosis of diabetes, was being treated with blood glucose lowering medications, or had a random blood glucose measurement of ≥200 mg/dL. Obesity was defined as a BMI of >30 kg/m2. Tobacco use was categorized as current smokers, past smokers or lifetime non-smokers.

We calculated the 10-year risk of a fatal/nonfatal cardiovascular event using the World Health Organization/International Society of Hypertension (WHO/ISH) risk charts calibrated for use in South-East Asia regions B (SEAR B) [17]. High CVD risk was defined as the presence of any of the following: coronary heart disease, stroke or other atherosclerotic disease; estimated 10-year CVD risk of ≥30%; or estimated 10-year CVD risk between 20% to 29% combined with a systolic blood pressure of >140 mmHg.

### Treatment

Treatment of high CVD risk was defined as self-reported use of antihypertension, lipid lowering and antiplatelet medicines.

### Statistical analysis

We presented continuous variables as a mean ± standard deviation and categorical variables as frequency and percent. Continuous variables between different areas (urban, semi-urban and rural) were compared using analysis of variance, while categorical variables were compared using chi-square tests. We conducted stratified analysis by residential area and classified the outcomes based on age, sex, educational level, marital status, employment status and the presence of obesity, with p-values for heterogeneity or trend presented. Multivariable logistic models were constructed to explore the associated variables to high 10-year cardiovascular risk. The treatment of high cardiovascular risk was identified by calculating proportions with their confidence intervals. For treatment, the numerator was the number of those who were receiving treatment, and the denominator was the number of those with high cardiovascular risk. All statistical analyses were performed using STATA version 14.

## Results

### Population characteristics

All adults aged ≥40 years in the eight participating villages were targeted in this study. Enumerators were able to screen 22,093 individuals representing 99.24% of the target population identified during census by independent field researchers (22,261). The mean age of the non-respondents was 42 and 61% of them were male. Table 1 illustrates the descriptive statistics of the 22,093 respondents and compares their characteristics according to residential area. A total of 10,209, 4,228, and 7,656 participants resided in urban, semi-urban and rural areas, respectively. The mean age of the respondents was 54.9 ± 10.7 years. On average, more than half of the respondents were female and had received a primary education or less. Respondents in the urban areas tended to have higher educational levels than those in semi-urban and rural areas; the proportion of those living in the urban areas with a college or higher educational level was almost four times greater than the proportion in the semi-urban and rural areas. In all areas combined, approximately 80% of respondents were married; slightly more than one-third of the respondents had no paid job. However, each area showed a different pattern of employment status. The employment status of the respondents consists of casual workers, government workers, private workers, self-employed, and not working. Casual workers are those working on an irregular or flexible basis, often to meet a fluctuating demand for work. The proportion of those who worked as casual workers was highest in rural areas, while that of private workers was highest in semi-urban areas (31.2%). Government work, self-employment and un-employment were more common in urban areas than in other areas.

**Table 1:** Characteristics of the participants.

	Total n = 22,093	Urban n = 10,209	Semi-urban n = 4,228	Rural n = 7,656	P-value
Age (years)	54.9 (10.7)	54.8 (10.5)	54.5 (10.5)	55.1 (11.2)	0.16
Age group					0.003
40–44	3,951 (17.8)	1,781 (17.4)	790 (18.6)	1,380 (18.0)	
45–49	4,227 (19.1)	1,960 (19.2)	794 (18.7)	1,473 (19.2)	
50–54	4,021 (18.2)	1,873 (18.3)	792 (18.7)	1,356 (17.7)	
55–59	3,328 (15.0)	1,584 (15.5)	647 (15.3)	1,097 (14.3)	
60–64	2,443 (11.0)	1,147 (11.2)	465 (11.0)	831 (10.8)	
65–69	1,689 (7.6)	799 (7.8)	309 (7.3)	581 (7.5)	
70–74	1,097 (4.9)	493 (4.8)	208 (4.9)	396 (5.1)	
75+	1,337 (6)	572 (5.6)	223 (5.2)	542 (7)	
Female	12,512 (56.6)	5,730 (56.1)	2,411 (57)	4,371 57)	0.37
*Educational level*					<0.001
Primary school or less	13,416 (60.7)	4,541 (44.4)	3,117 (73.7)	5,758 (75.3)	
Secondary school	7,314 (33.1)	4,630 (45.3)	999 (23.6)	1,658 (22.0)	
College or higher	1,350 (6.1)	1,038 (10.1)	111 (2.6)	201 (2.6)	
*Marital status*					<0.001
Single	351 (1.5)	223 (2.1)	31 (0.7)	97 (1.2)	
Married	17,989 (81.6)	8,216 (80.7)	3,494 (82.7)	6,279 (82.3)	
Divorced/separated	614 (2.7)	290 (2.8)	106 (2.5)	218 (2.8)	
Widowed	3,076 (13.9)	1,451 (14.2)	593 (14.0)	1,032 (13.5)	
*Employment status*					<0.001
Casual workers	4,225 (19.1)	998 (9.7)	618 (14.6)	2,609 (34.0)	
Government workers	781 (3.5)	517 (5)	115 (2.7)	149 (1.9)	
Private workers	4,222 (19.1)	1,799 (17.6)	1,319 (31.2)	1,104 (14.4)	
Self-employed	5,221 (23.6)	3,119 (30.5)	734 (17.3)	1,368 (17.8)	
Not working	7,637 (34.5)	3,772 (36.9)	1,441 (34)	2,424 (31.6)	
Mean systolic BP (mmHg)	140.3 (23.7)	140.7 (24.3)	140.8 (23.8)	139.6 (22.9)	0.03
Mean diastolic BP (mmHg)	88.4 (13.0)	88.9 (13.2)	88.7 (13.1)	87.5 (12.6)	<0.001
BMI (kg/m^2^)	24.9 (4.7)	25.1 (4.6)	25.2 (4.7)	24.5 (4.7)	<0.001
Random plasma glucose (mg/dL)	126 (60.4)	125.8 (61.8)	127.6 (61.6)	125.3 (57.9)	<0.001
*Smoking behavior*					<0.001
Current smokers, n(%)	5,880 (26.6)	2,563 (25.1)	1,156 (27.3)	2,161 (28.2)	
Past smokers, n(%)	2,140 (9.6)	1,057 (10.3)	455 (10.7)	628 (8.2)	
Non-smokers, n(%)	14,073 (63.7)	6,589 (64.5)	2,617 (61.9)	4,867 (63.5)	
*10-year risk of CVD*					<0.001
<10%	14,500 (65.6)	6,485 (63.5)	2,783 (65.8)	5,232 (68.3)	
10–20%	1,131 (5.1)	493 (4.8)	229 (5.4)	409 (5.3)	
20–30%	214 (0.9)	105 (1.0)	34 (0.8)	75 (0.9)	
30–40%	13 (0.06)	4 (0.04)	4 (0.09)	5 (0.07)	
<40%	7 (0.03)	5 (0.05)	1 (0.02)	1 (0.01)	
Past history of CVD	765 (3.4)	435 (4.2)	125 (2.9)	205 (2.6)	
Clinical high-risk	5,461 (24.7)	2,681 (26.2)	1,051 (24.7)	1,729 (22.5)	

The mean BP overall was 140.3/88.4 mmHg, with a significant trend of lower levels with higher degree of rurality. More than one-quarter of the respondents in all three areas (26.6%) were active smokers, but this proportion was greatest in rural areas and lowest in urban areas.

### Prevalence of cardiovascular risk

**Fig 2:**
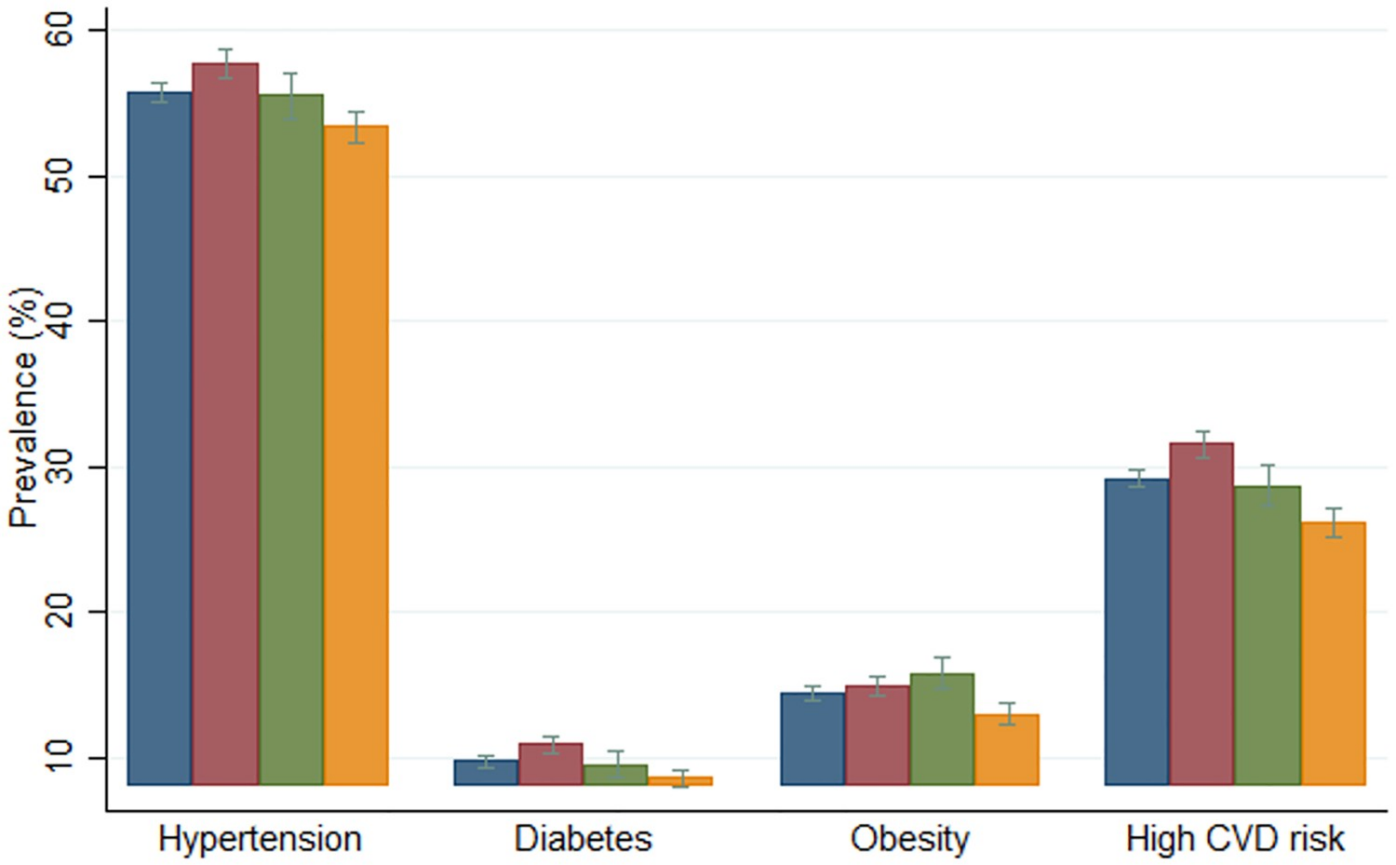
Prevalence of cardiovascular risk factors and 10-year cardiovascular risk. Colours indicate the areas: blue represents total areas, red represents urban areas, green represents semi-urban areas, and yellow represents rural areas.

Fig 2 shows the prevalence of cardiovascular risk factors (hypertension, diabetes and obesity) and high 10-year cardiovascular risk in total and by rurality. Hypertension was found to be the most common cardiovascular risk factor (55.8%), followed by obesity (14.4%), with an overall prevalence of diabetes of 9.8% of the population. The prevalence of hypertension and diabetes was highest in urban areas, followed by semi-urban and rural areas. In contrast, obesity was most prevalent in semi-urban areas. The prevalence of high 10-year cardiovascular risk was highest in urban areas (31.6%, CI 30.7–32.5%) and lowest in rural areas (26.2%, CI 25.2–27.2%).

The prevalence of high 10-year cardiovascular risk was higher among those in older age groups, and the difference was more marked among those living in urban areas (Table 2). The prevalence of high 10-year cardiovascular risk was higher among females in urban areas than in semi-urban and rural areas. Lower levels of educational attainment were associated with greater prevalence of high 10-year cardiovascular risk in all residential areas. The prevalence of high 10-year cardiovascular risk was greatest among widowed respondents in all areas. The patterns of high 10-year cardiovascular risk among respondents with different employment status vary by residential area. Although the greatest prevalence of high 10-year cardiovascular risk in all areas was found among those without any paid job, the prevalence was lowest among private workers in urban (22%) and semi-urban (22.5%) areas and among government workers in rural areas (17.4%). In all areas, the prevalence of high 10-year cardiovascular risk was greater among the obese.

**Table 2:** Prevalence of high 10-year cardiovascular risk among the populations in urban, semi-urban, and rural areas by education, marital status, employment status, physical activity level and BMI.

	Total		Urban		Semi-urban		Rural	
	%	CI*	%	CI*	%	CI*	%	CI*
*Age group*								
40–44	14.7	13.5–15.8	15.1	13.4–16.8	13.7	11.2–16.2	14.7	12.8–16.6
45–49	21.5	20.2–22.7	22.8	20.9–24.7	22.5	19.6–25.6	19.1	17.0–21.1
50–54	28.4	27.0–29.8	30.6	28.5–32.7	30.2	26.9–33.5	24.4	22.1–26.7
55–59	31.1	29.4–32.6	34.3	32.0–36.7	30.8	27.2–34.4	26.5	23.9–29.2
60–64	38.2	36.2–40.1	42.1	39.2–45.0	34.8	30.5–39.3	34.7	31.4–38.0
65–69	39.5	37.1–41.8	43.7	40.2–47.1	40.8	35.2–46.4	33.0	29.2–37.0
70–74	47.7	44.6–50.6	50.9	46.4–55.4	47.1	40.1–54.1	43.9	38.9–48.9
75+	49.8	47.0–52.5	54.5	50.3–58.6	46.2	39.5–52.9	46.3	42.0–50.6
*Sex*								
Male	26.3	25.3–27.1	26.3	28.2–30.8	25.1	23.1–27.1	22.4	21.0–23.9
Female	31.5	30.6–32.2	31.5	32.0–34.4	31.4	29.5–33.3	29.1	27.8–30.5
*Educational level*								
Primary school or less	31.3	30.4–32.0	31.3	34.5–37.3	30.8	29.2–32.4	27.9	26.7–29.0
Secondary school	26.0	25.0–27.0	26.0	26.9–29.5	22.7	20.1–25.4	21.9	19.9–23.9
College or higher	25.9	23.6–28.3	25.9	25.1–30.6	23.4	15.9–32.4	17.4	12.4–23.3
*Marital status*								
Single	22.2	17.9–26.9	22.2	18.7–30.3	22.6	9.5–41.0	17.5	10.5–26.5
Married	27.1	26.5–27.8	27.1	28.4–30.4	26.7	25.2–28.2	24.3	23.2–25.4
Divorced/separated	30.8	27.1–34.6	30.8	29.0–40.2	27.4	19.1–36.8	27.5	21.7–33.9
Widowed	41.8	40.0–43.5	41.8	41.8–47	41.0	36.9–45	38.5	35.4–41.5
*Employment status*								
Casual workers	23.0	21.7–24.2	23.0	23.0.28.6	23.9	20.6–27.5	21.7	20.0–23.2
Government workers	24.3	21.3–27.4	24.3	22.9–30.7	22.6	15.3–31.3	17.4	11.7–24.5
Private workers	22.0	20.7–23.2	22.0	21.3–25.3	22.5	20.2–24.8	19.1	16.8–21.5
Self-employed	28.2	26.9–29.4	28.2	27.7–30.9	27.1	23.9–30.4	26.2	23.8–28.5
Not working	37.8	36.7–38.9	37.8	38.0–41.2	37.8	35.2–40.3	35.1	33.1–37
*Obesity*								
Not present	27.6	26.9–28.2	27.6	29.1–31.0	27.3	25.8–28.7	24.5	23.4–25.5
Present	38.8	37.1–40.5	38.8	37.6–42.6	36.4	32.7–40.1	38.3	35.3–41.4

Note:

* CI = 95% Confidence intervals

On multivariable logistic regression analysis, the covariates of high 10-year cardiovascular risk were age, area of residence, employment status and the presence of obesity (Table 3). Table 3 shows that respondents in semi-urban and urban areas had higher odds of having high cardiovascular risk than those in rural areas. Those who were employed as government workers, private sector workers, self-employed, and unemployed were more likely to have high cardiovascular risk than casual workers. Obese respondents had an 85% greater probability of having high cardiovascular risk.

**Table 3:** Associated variables to high 10-year cardiovascular risk.

	COR (CI 95%)	AOR (CI 95%)
*Age group*		
40–44	Reference	Reference
45–49	1.58 (1.41–1.78)***	1.62 (1.44–1.82)***
50–54	2.3 (2.06–2.58)***	2.4 (2.14–2.69)***
55–59	2.61 (2.33–2.93)***	2.72 (2.41–3.06)***
60–64	3.59 (3.18–4.04)***	3.7 (3.26–4.2)***
65–69	3.79 (3.32–4.32)***	3.92 (3.4–4.51)***
70–74	5.29 (4.56–6.13)***	5.3 (4.51–6.22)***
75+	5.76 (5.02–6.62)***	5.5 (4.72–6.48)***
*Area*		
Rural	Reference	Reference
Semi-urban	1.13 (1.03–1.22)***	1.09 (1–1.2)**
Urban	1.29 (1.21–1.38)***	1.23 (1.14–1.32)***
*Sex*		
Male	Reference	Reference
Female	1.28 (1.21–1.36)***	1.06 (0.98–1.14)
*Educational level*		
Primary school or less	Reference	Reference
Secondary school	0.77 (0.72–0.82)***	0.97 (0.9–1.05)
College or higher	0.76 (0.67–0.87)***	0.93 (0.9–1.05)
*Marital status*		
Single	Reference	Reference
Married	1.3 (1.01–1.68)*	1.01 (0.78–1.32)
Divorced/separated	1.55 (1.14–2.11)***	1.05 (0.76–1.44)
Widowed	2.51 (1.93–3.26)***	1.04 (0.79–1.37)
*Employment status*		
Casual worker	Reference	Reference
Government worker	1.07 (0.9–1.28)	1.23 (1–1.51)**
Private worker	0.94 (0.85–1.04)	1.18 (1.06–1.32)***
Self-employed	1.31 (1.19–1.44)***	1.33 (1.2–1.47)***
Not working	2.03 (1.87–2.22)***	1.64 (1.49–1.81)***
*Obesity*		
Not present	Reference	Reference
Present	1.66 (1.53–1.79)***	1.85 (1.7–2.01)***

**Notes:** COR: Crude Odds Ratio; AOR: Adjusted Odds Ratio; CI 95% Confidence intervals 95%.

Significance:

* p<0.05;

** p<0.01;

***p<0.001

### Treatment of hypertension

Table 4 presents preventive treatments among individuals with high CVD risk in rural, semi-urban, and urban areas. Of all the respondents with high CVD risk (6453), 774 and 95 were on blood pressure lowering and statins treatment, respectively. Only 17 of them received optimal preventive treatment (combination between blood pressure lowering treatment, statins treatment and antiplatelet). Residents of urban areas were more likely to be receiving blood pressure lowering and statins treatment than residents of semi-urban and rural areas, and the difference was statistically significant (p<0.001). However, those significant differences did not appear on the optimal preventive treatment.

**Table 4:** Preventive treatments among individuals with high CVD risk in rural, semi-urban, and urban areas.

		On blood pressure lowering treatment	On statins treatment	On optimal preventive treatment
Total	n/N	774/6453	95/6453	17/6453
	%	11.99	1.47	0.26
	CI	11.21/12.81	1.19/1.79	0.15/0.42
Urban	n/N	485/3228	65/3228	12/3228
	%	15.02	2.01	0.37
	CI	13.80/16.30	1.55/2.55	0.19/0.64
Semi-urban	n/N	135/1214	11/1214	3/1211
	%	11.15	0.91	0.25
	CI	9.40/13.02	0.45/1.61	0.05/0.72
Rural	n/N	154/2011	19/2011	2/2011
	%	7.66	0.94	0.10
	CI	6.53/8.90	0.56/1.47	0.01/0.35
P-value		<0.001	0.001	0.173

n: number with condition (numerator); N: denominator

## Discussion

This study is the first large, cross-sectional, population-based study to address the prevalence of the 10-year risk of CVD using the WHO/ISH scoring system [17] in Indonesia. We found that high CVD risk was very common in Malang district, with almost 30% of the adult population aged 40 years and above affected. With respect to other studies that used the WHO/ISH risk scores, the prevalence of high 10-year cardiovascular risk that we found in Indonesia is higher than those of rural India (10.2%) with the same age range of sample (40 years and older [18]. A study with respondents aged 40 until 64 years old in three countries shows that the prevalence of high CVD risk in Mongolia (33.3%) is similar with our study, and those in Cambodia (10.4%) and Malaysia (20.8%) were lower than our study [19].

In this study, the prevalence of high CVD risk was greatest among residents of urban villages, followed by those in semi-urban and rural villages. While there is a statistically significant difference in these prevalence rates, the absolute differences are very small. Assuming there are more people who live in rural areas, the absolute numbers of high risk people are greatest in rural areas. A higher prevalence of CVD risk among urban populations than their rural counterparts has been previously observed throughout the world [8, 20–22]. Our findings suggest that increasing life expectancy and urbanisation are major determinants of CVD in developing countries. Urbanisation has been associated with higher CVD incidence [23, 24], including in South East Asian countries [25]. More than half of Indonesians (55%) were living in urban areas in 2010, and this proportion is projected to increase to 66% by 2035 [26]. In additional to nutritional issues, tobacco smoking is an important risk factor for cardiovascular diseases. Our study found that 26.6% and 9.6% of Indonesian adults aged 40 years and older were current smokers and past smokers, respectively. Using 2007 Basic Health Survey data, a prior study found that current smokers who had smoked for less than 20 years had higher odds of having CVD (OR = 1.43; CI 1.17–1.76) than non-smokers. Past smokers were twice more likely to suffer from CVD than non-smokers [27]. Although Indonesia is one of the largest consumers of tobacco worldwide, little has been done to reduce the rates of tobacco addiction in the country. Identifying appropriate population-based strategies for tobacco control, such as WHO best buys, should be a top priority for policy makers in Indonesia, given the high rates of tobacco use.

Our study shows several important findings regarding the treatment received by the respondents with high cardiovascular risk in Indonesia. We found that only 11% and 1% of them received blood pressure lowering and statins treatment. Furthermore, less than 1 in 370 respondents with high CVD risk were on optimal preventive treatment.

These findings show the urgent need to primary healthcare system strengthening to address this need. The proportion of respondents receiving the treatment was higher in urban areas. These findings reinforce the evidence that, notwithstanding the efforts of the government to increase healthcare access, inequality between urban and rural areas still persist where cardiovascular care is concerned.

As cardiovascular risk is largely asymptomatic, the capacity of the health system to provide information and diagnostic services to the population is crucial for awareness of cardiovascular risk. The Ministry of Health has initiated *Posbindu* to promote community participation in the prevention, early detection, and monitoring of risk factors for non-communicable diseases [9]. A prior study, however, demonstrated that only 10% of the local population were using *Posbindu* services; educational levels, knowledge about *Posbindu*, health perception, and the charging of an administration fee are among the determinants of *Posbindu* utilization in South Jakarta [12]. A study in three districts in East Java province supported those findings and found that the poor access to the *Posbindu*, lack of support, especially from the family and physical impairment are among the barrier for older adults seeking healthcare services in *Posbindu* [28]. Continued sharing of knowledge about the role of *Posbindu* as well as about the importance of early detection and continuous monitoring of cardiovascular risk is needed to ensure that particularly older people acquire and maintain an awareness of the advantages of visiting *Posbindu*.

This study has a number of limitations. Firstly, some of our data were based on self-reported information. Although the interviews were carried out by well-trained personnel, the potential for bias was present (e.g. over- or underdiagnoses due to recall issues or subjectivity in the reporting of symptoms). Another limitation is that this research was based on residents in the district of Malang only, which might not represent the wider population in other districts and provinces in Indonesia. This study has several strengths. It used large data that is representative of the whole population. Another strength is the high response rate. Only 168 among 22,261 population aged 40 years and older in eight participating villages were not included in this research. Finally, it used standardised data collection tools. For example, the interviews and physical examination were performed by trained enumerators. The way they measured height, weight and blood pressure follows a standard protocol [13, 14].

## Conclusions

High cardiovascular risk is common in Malang District, with almost 30% of adults aged 40 years and older at high 10-year risk of developing CVD. However, only 24% of those at high risk were receiving treatment. Increasing awareness of CVD risk and screening programmes are needed to reduce the prevalence of CVD events. Furthermore, providing primary preventive approaches to ensure that people with low CVD risk retain that status should be a focus for policy-makers at the national level.

## Supporting information

S1 Table Characteristics of the villages. (DOCX)

S1 Appendix SMARTHealth Indonesia baseline study questionnaire. (DOCX)
