# Supplementary material for: Cardiovascular disease risk factor prevalence and estimated 10-year cardiovascular risk scores in Indonesia: The SMARThealth Extend study
Source: PLoS One. 2019 Apr 30;14(4):e0215219. doi: 10.1371/journal.pone.0215219 (PMC6490907; doi:10.1371/journal.pone.0215219)
Supplement: S1 Table — (DOCX) [file pone.0215219.s001.docx]

**S1 Table**. Characteristics of the villages

| Village name | Urban/rural | Major occupation(s) | Proximity to tobacco factories | Area in km^2^ | Population density | Gender ratio |
| --- | --- | --- | --- | --- | --- | --- |
| Karangduren | urban | business | very close | 5.10 | 1456 | 0.97 |
| Kendalpayak | urban | business | very close | 5.70 | 1778 | 0.98 |
| Kepanjen | urban | business | close | 1.65 | 7190 | 1.04 |
| Cepokomulyo | urban | business | close | 1.24 | 5704 | 1.09 |
| Sidorahayu | semi-urban | tobacco factory work | very close | 4.42 | 1991 | 0.98 |
| Mendalanwangi | semi-urban | tobacco factory work | very close | 3.58 | 2114 | 0.97 |
| Sepanjang | rural | farming | far | 10.00 | 1331 | 0.98 |
| Majangtengah | rural | farming | far | 8.13 | 1042 | 1.03 |
