# Supplementary figures and images for: Cardiovascular disease risk factor prevalence and estimated 10-year cardiovascular risk scores in Indonesia: The SMARThealth Extend study
Source: PLoS One. 2019 Apr 30;14(4):e0215219. doi: 10.1371/journal.pone.0215219 (PMC6490907; doi:10.1371/journal.pone.0215219)

SMART*health* India baseline study questionnaire


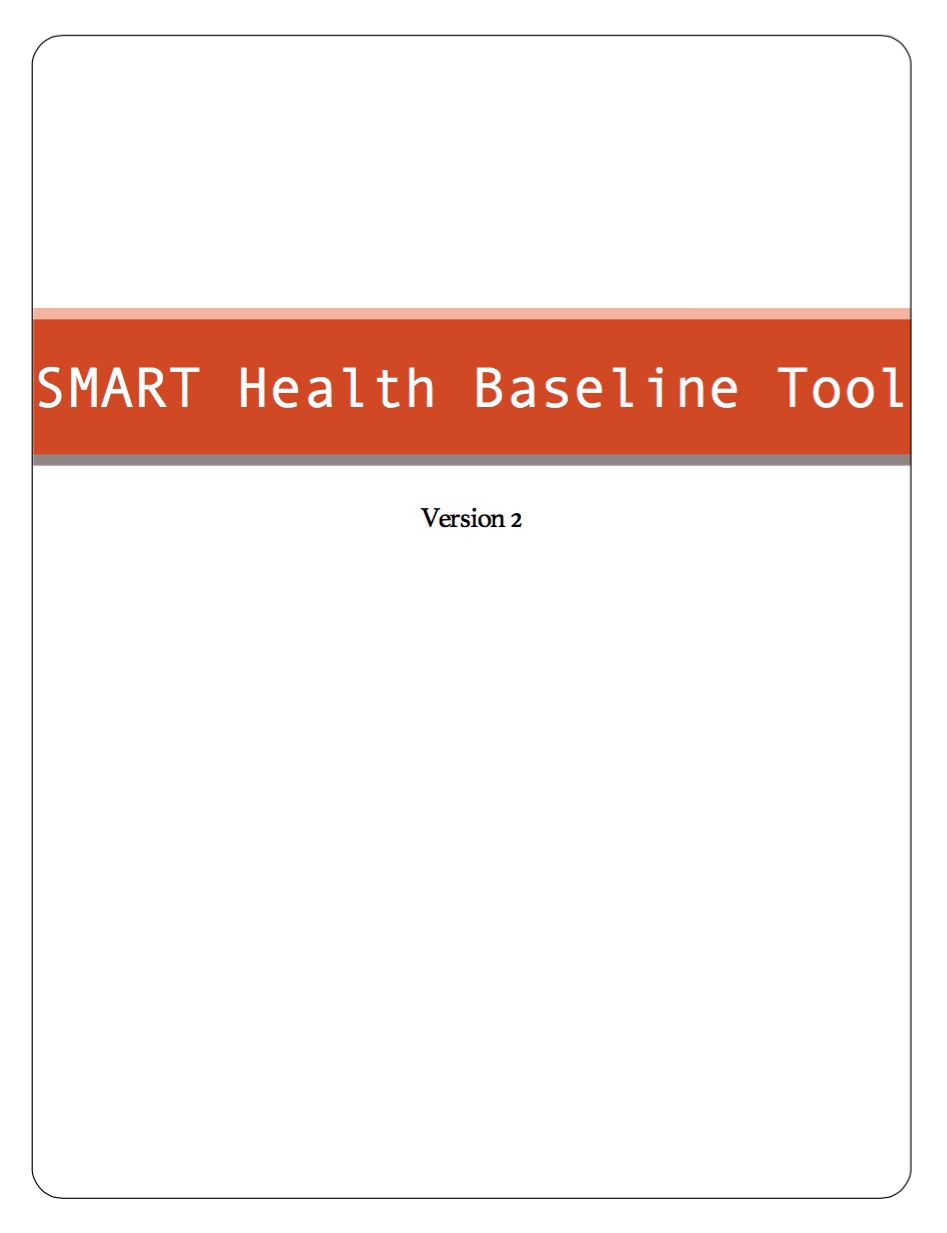


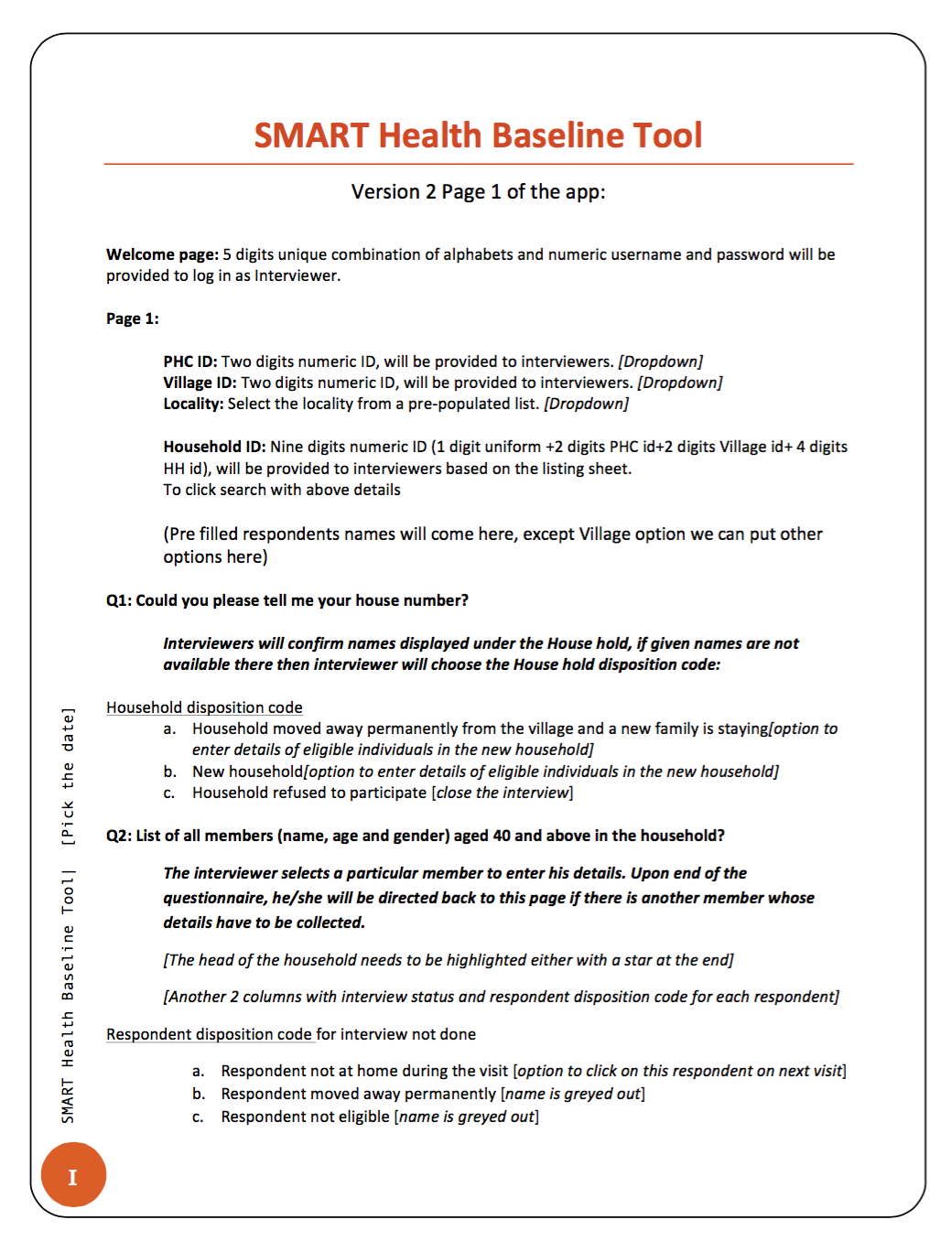


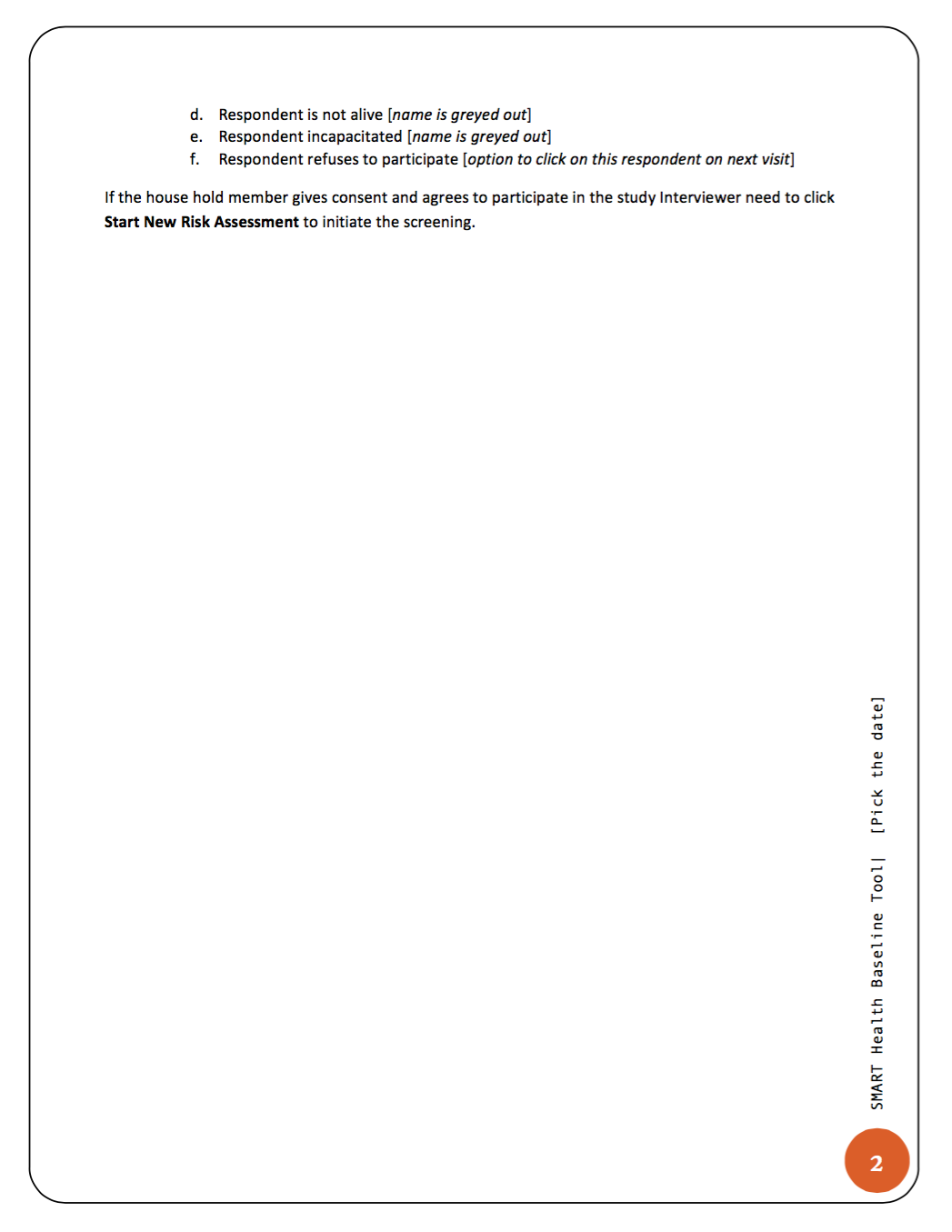


**
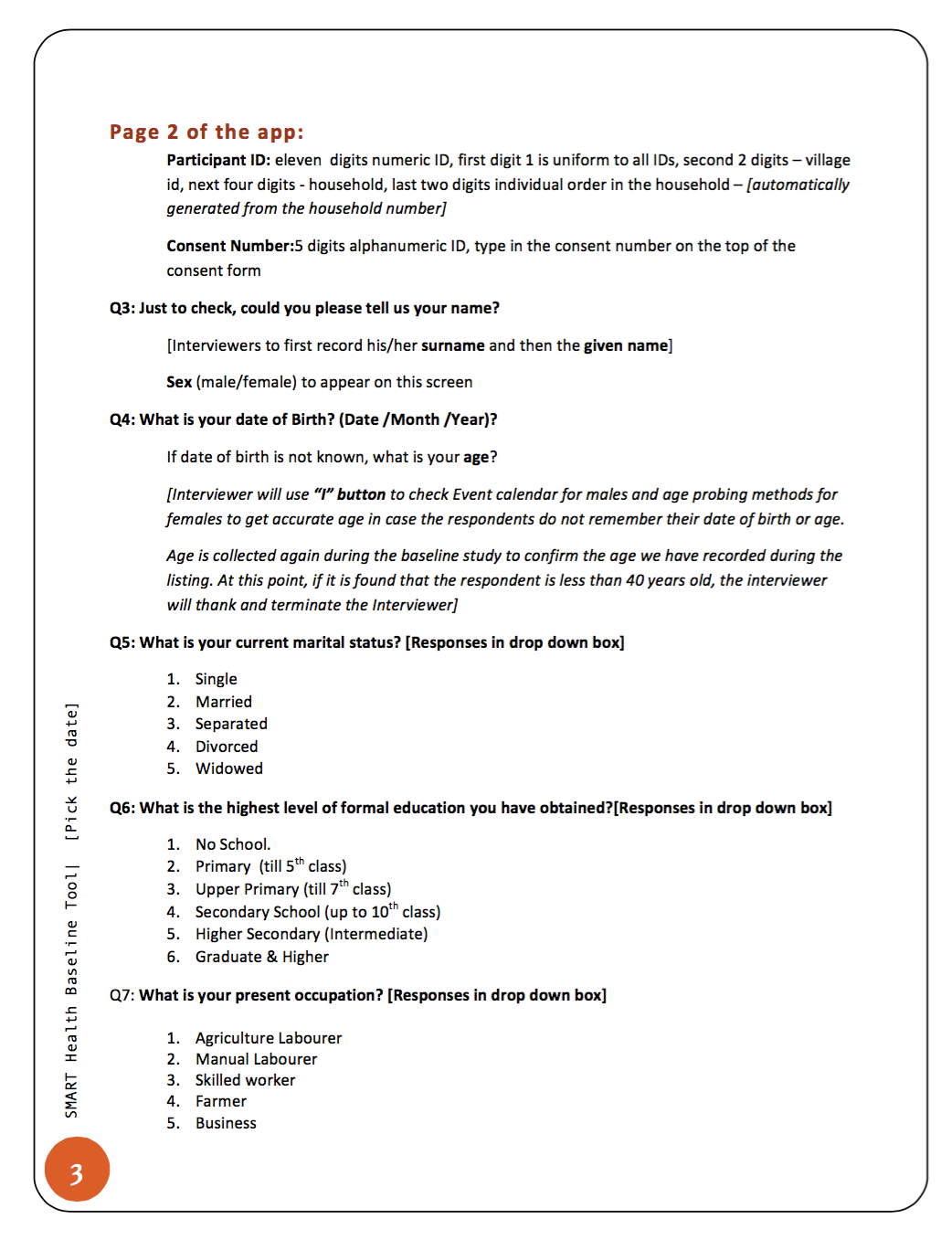
**


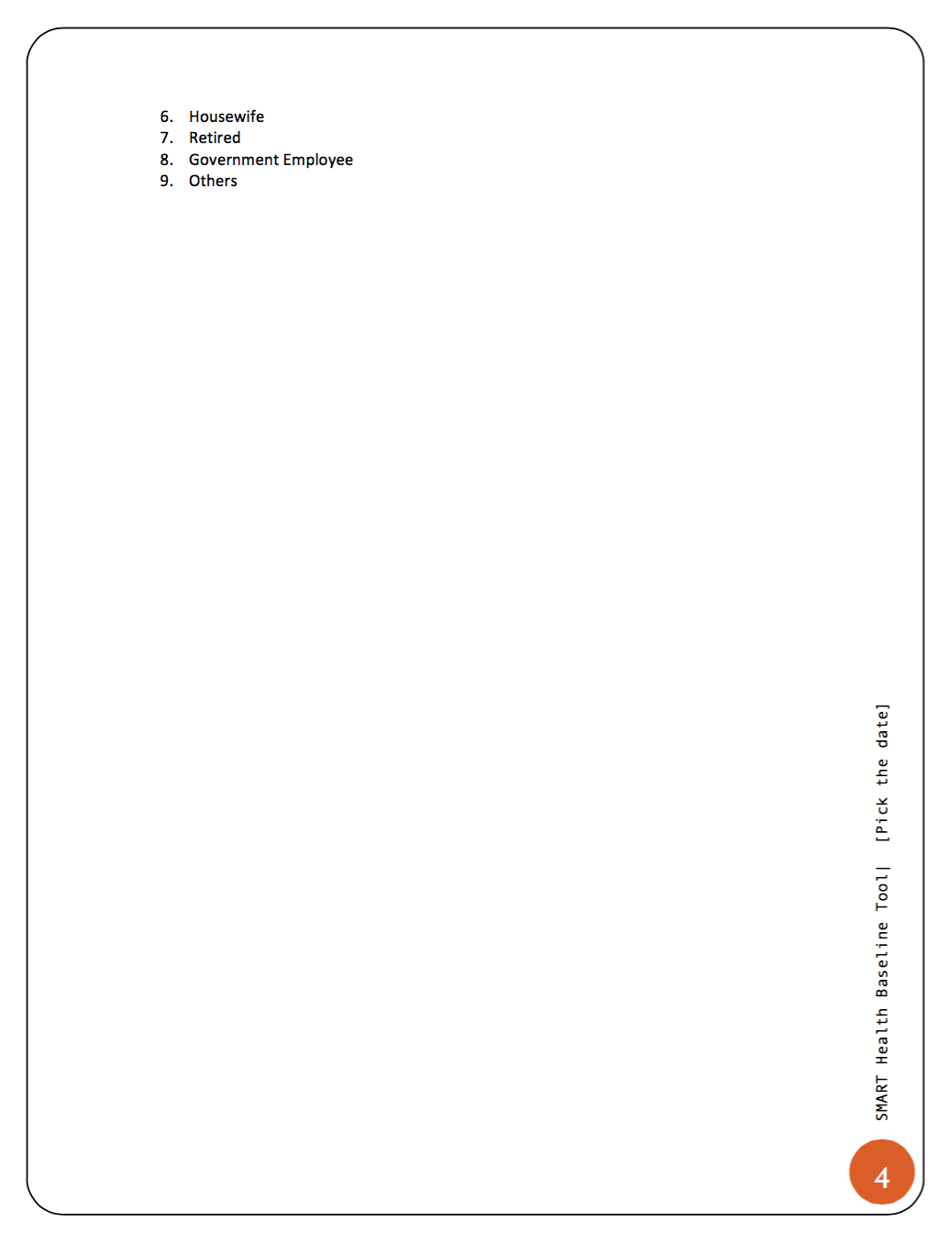


**
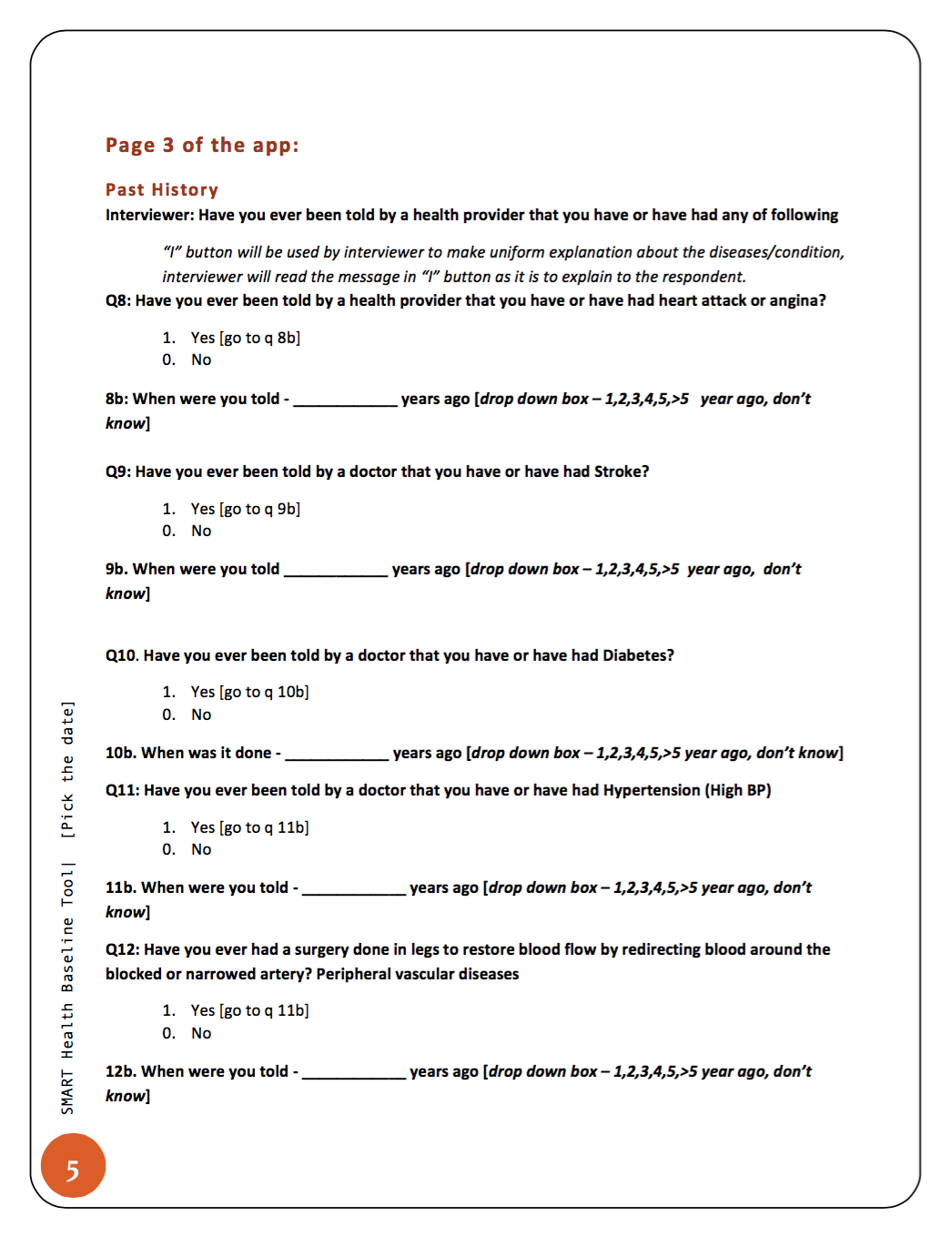
**


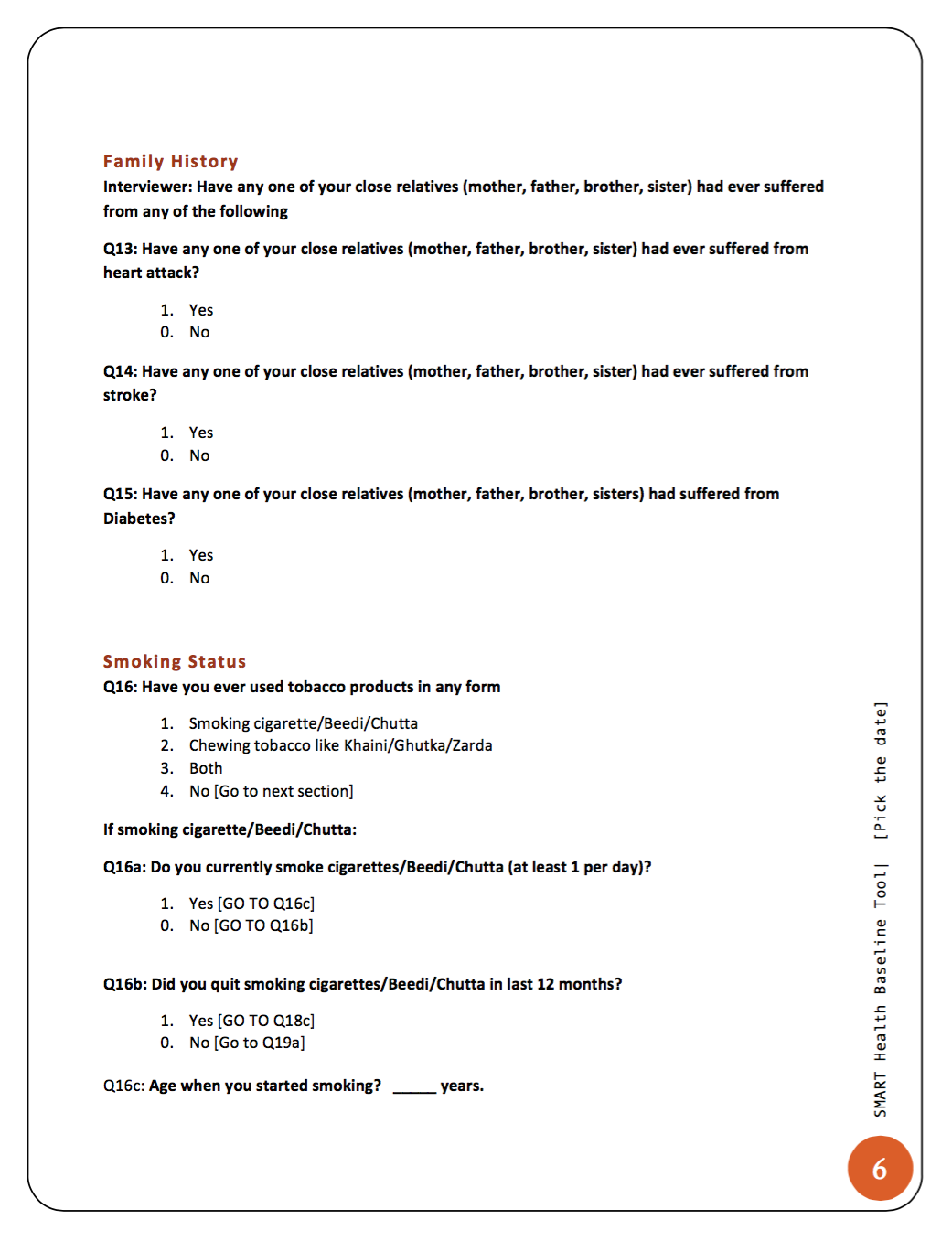


**
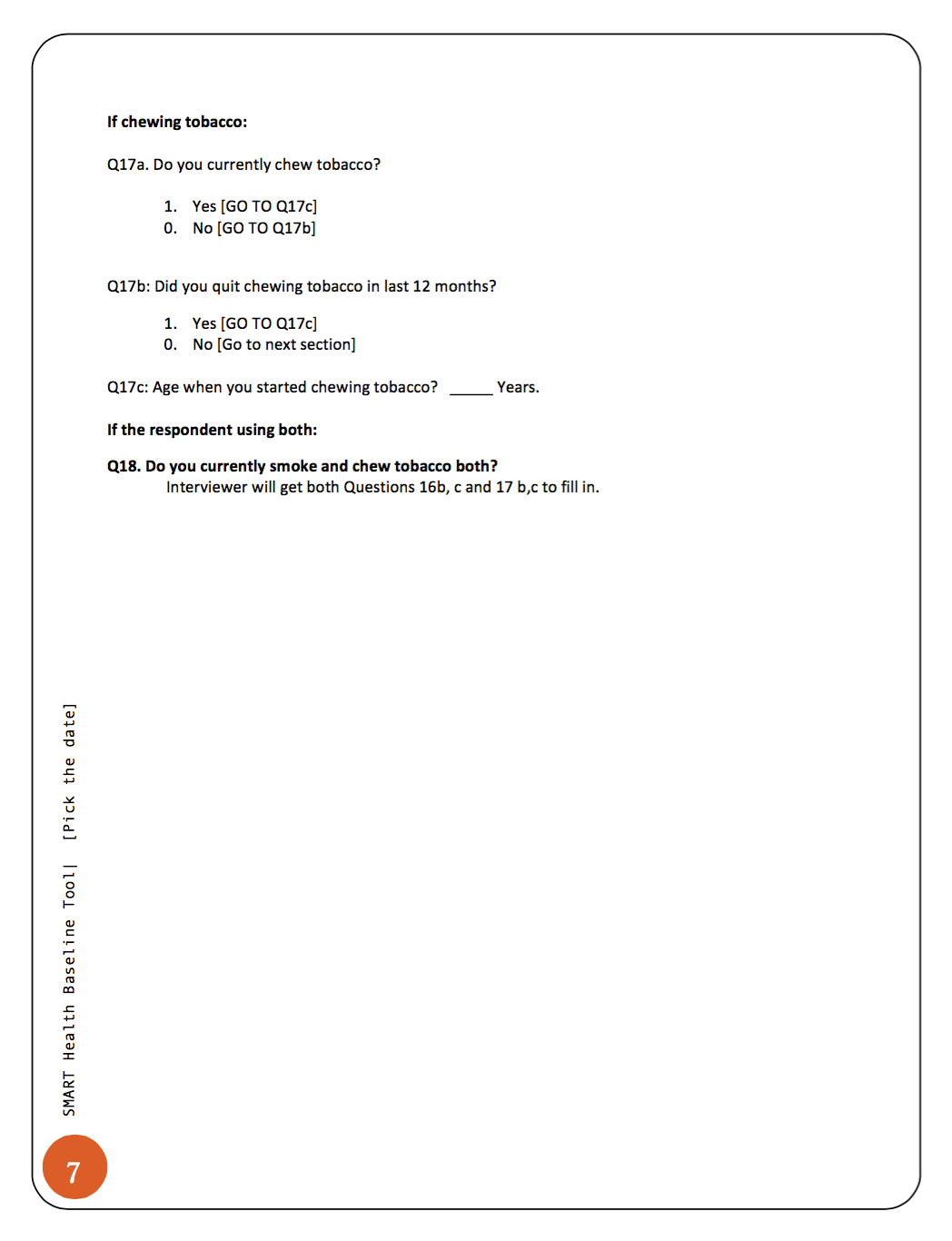
**


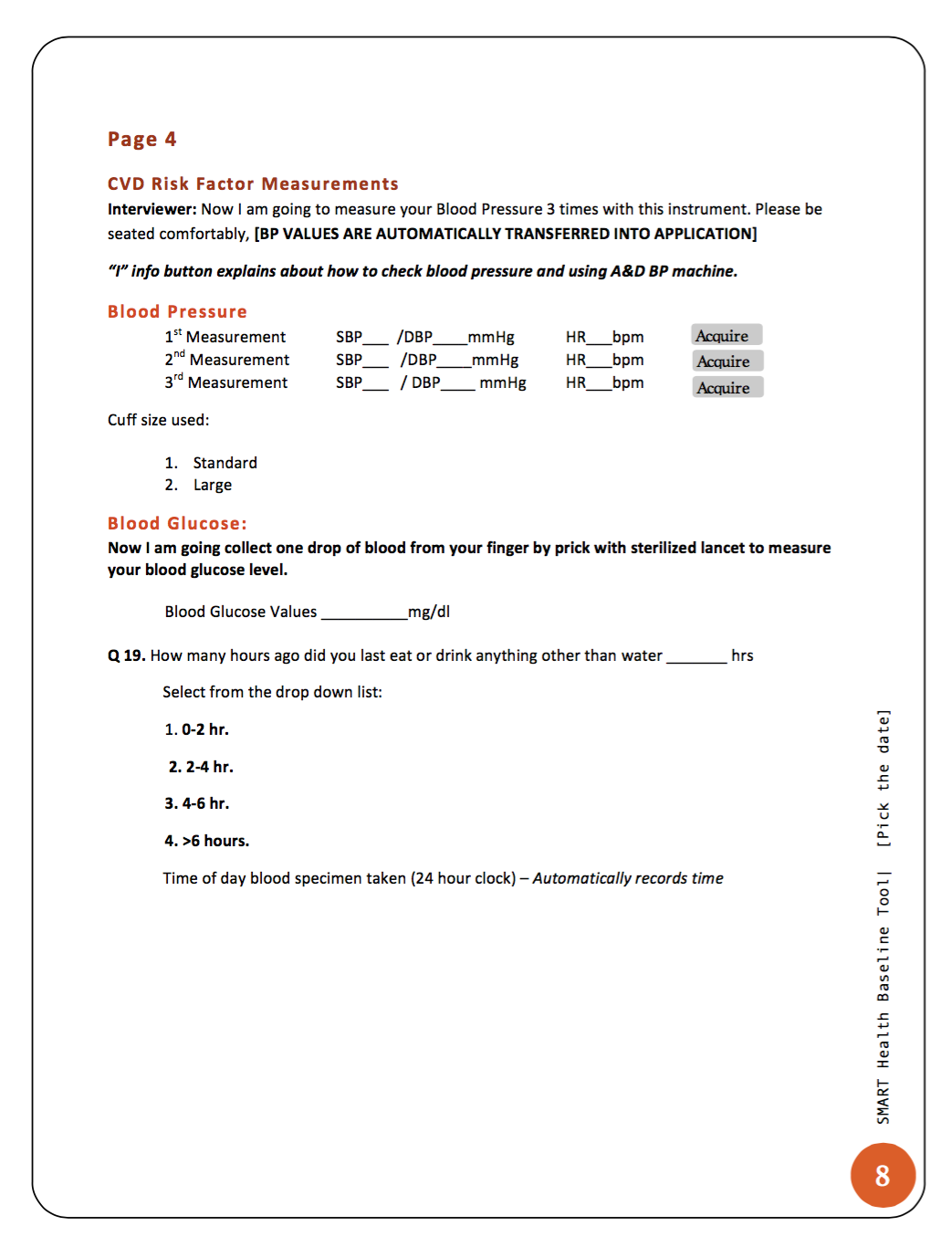


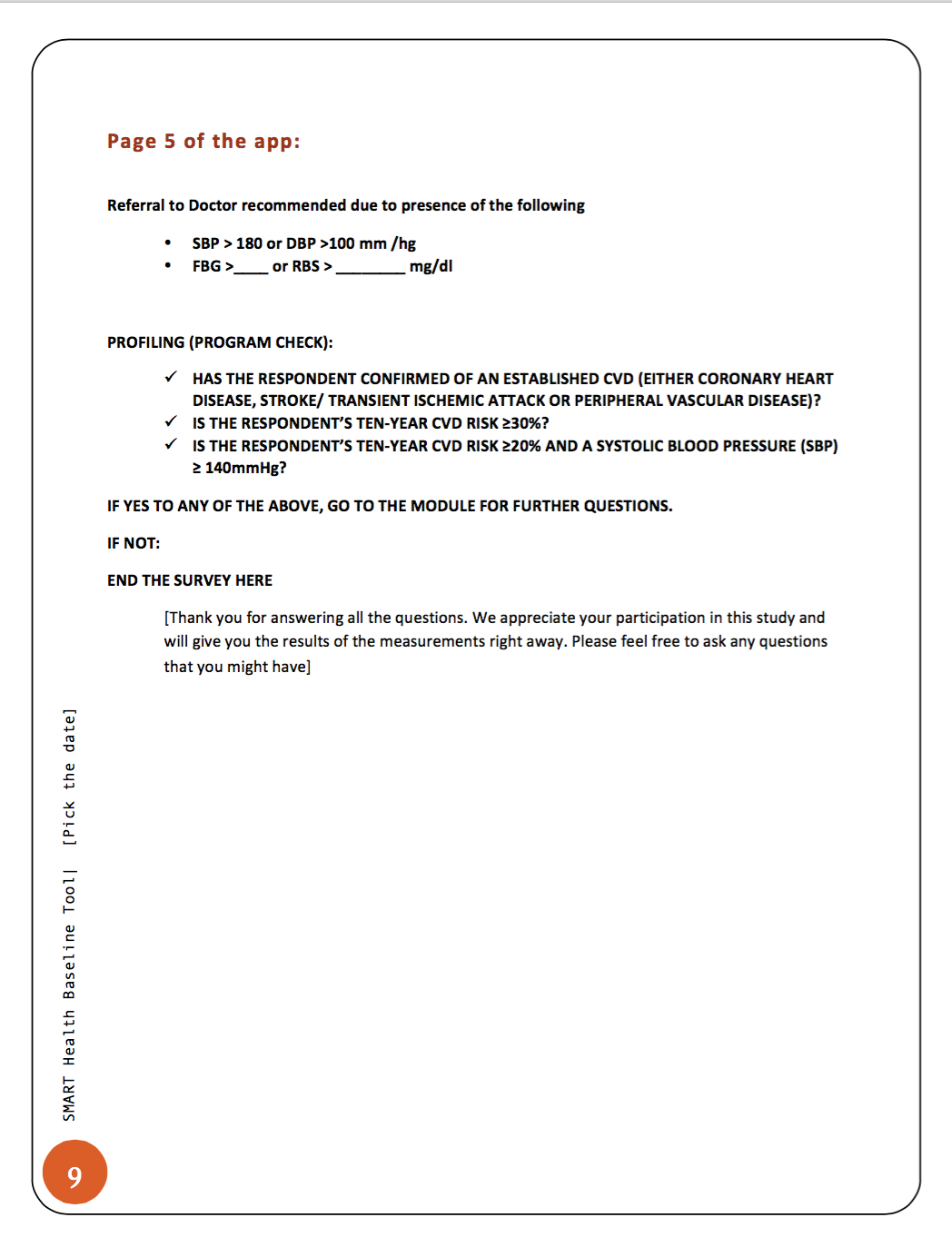


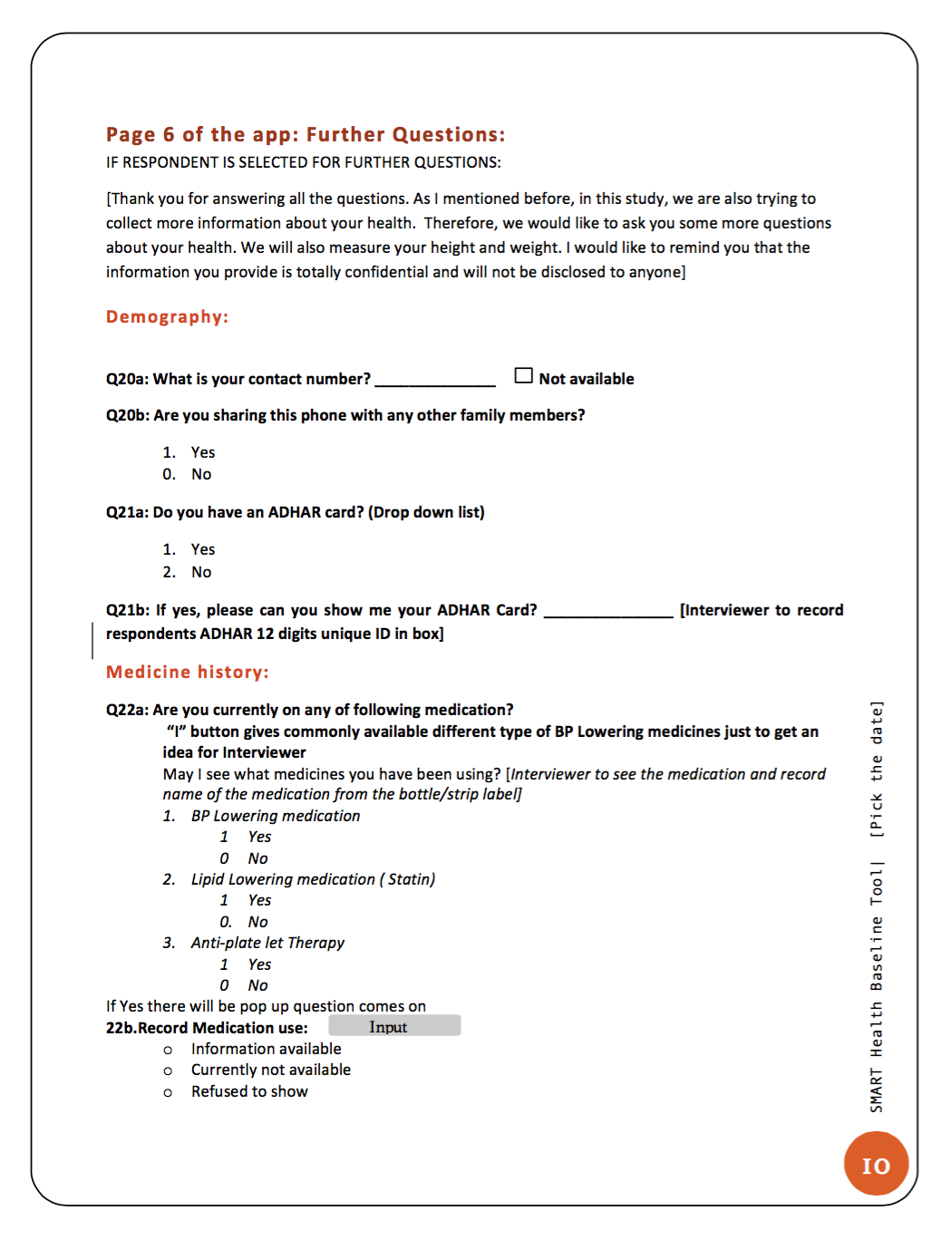

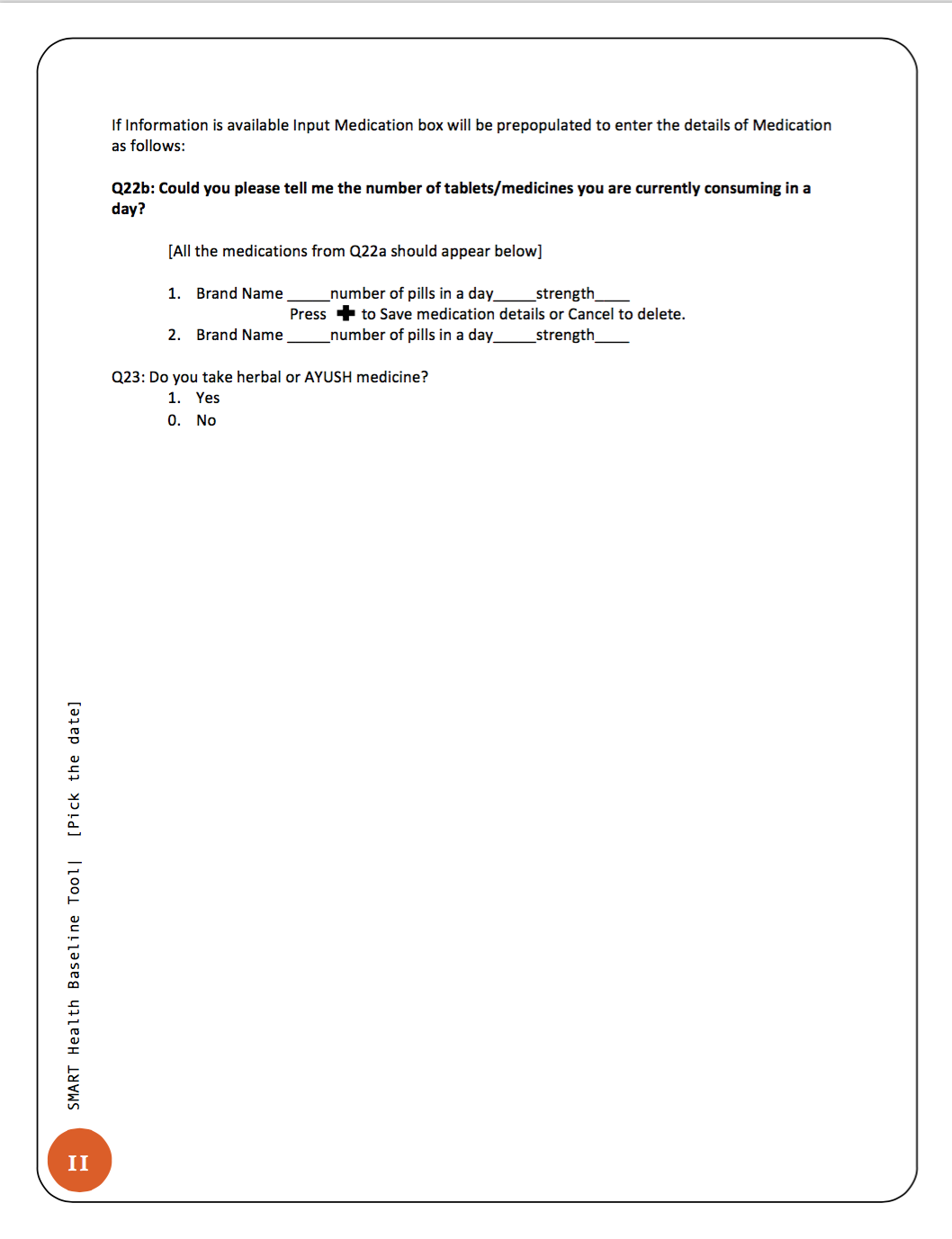


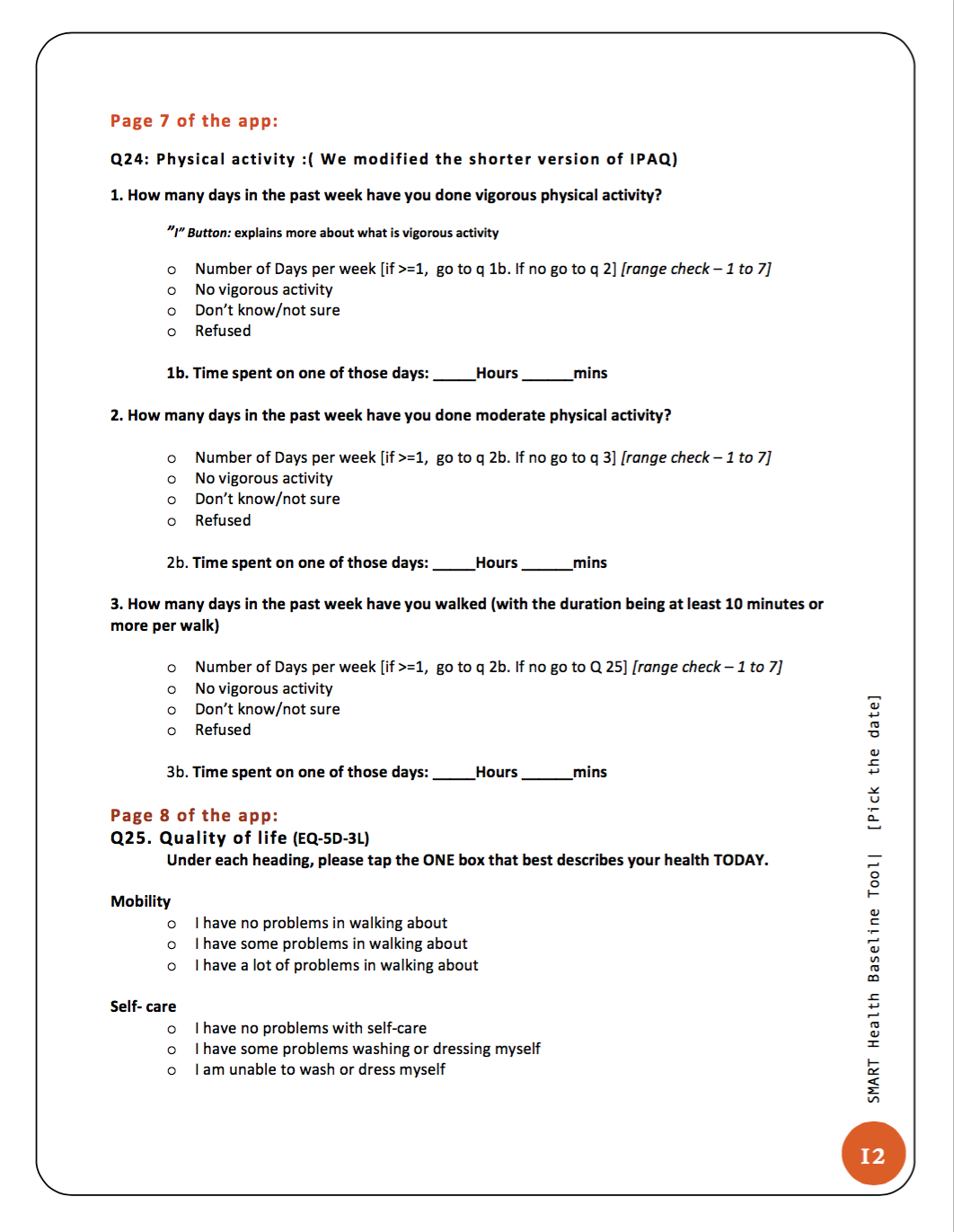


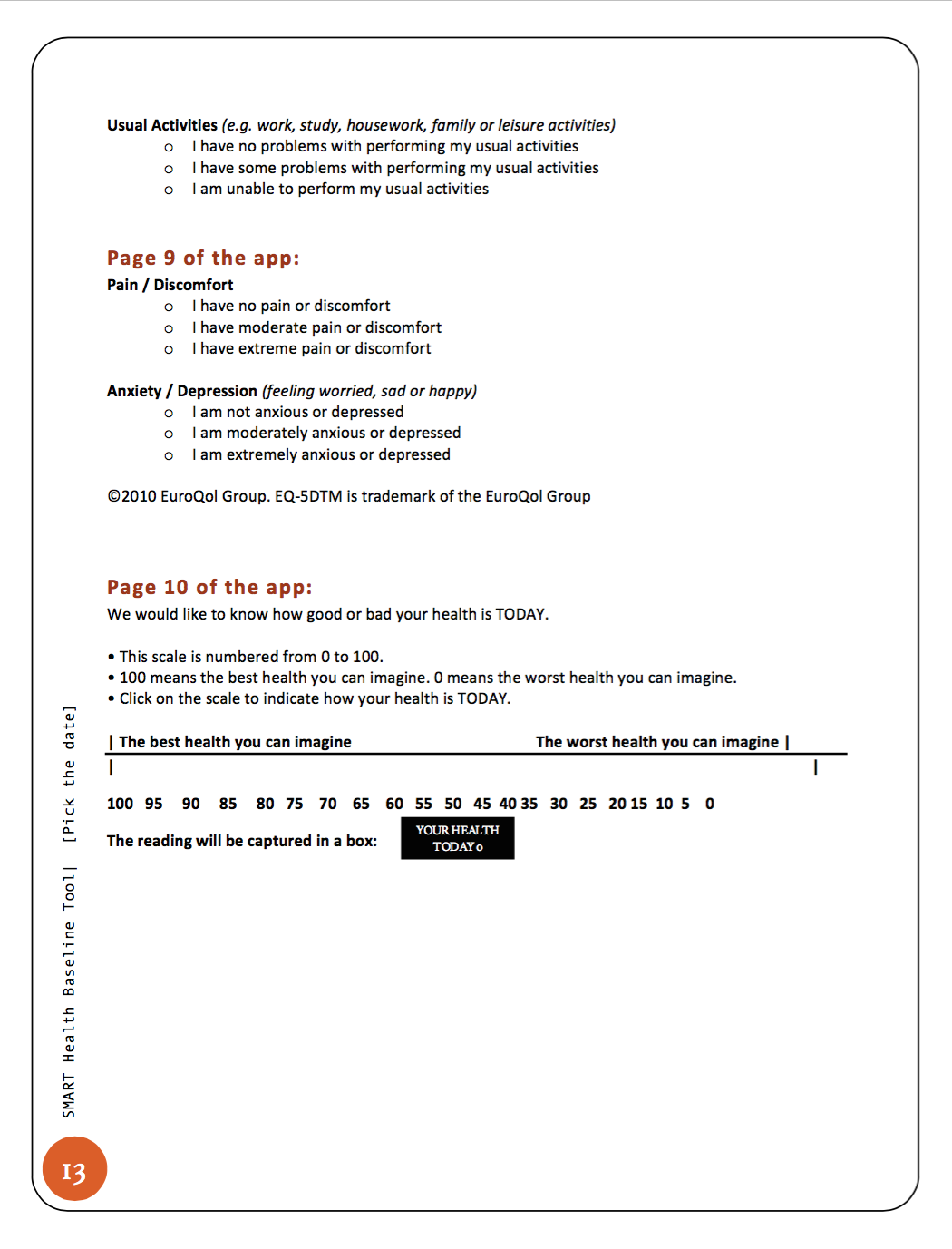


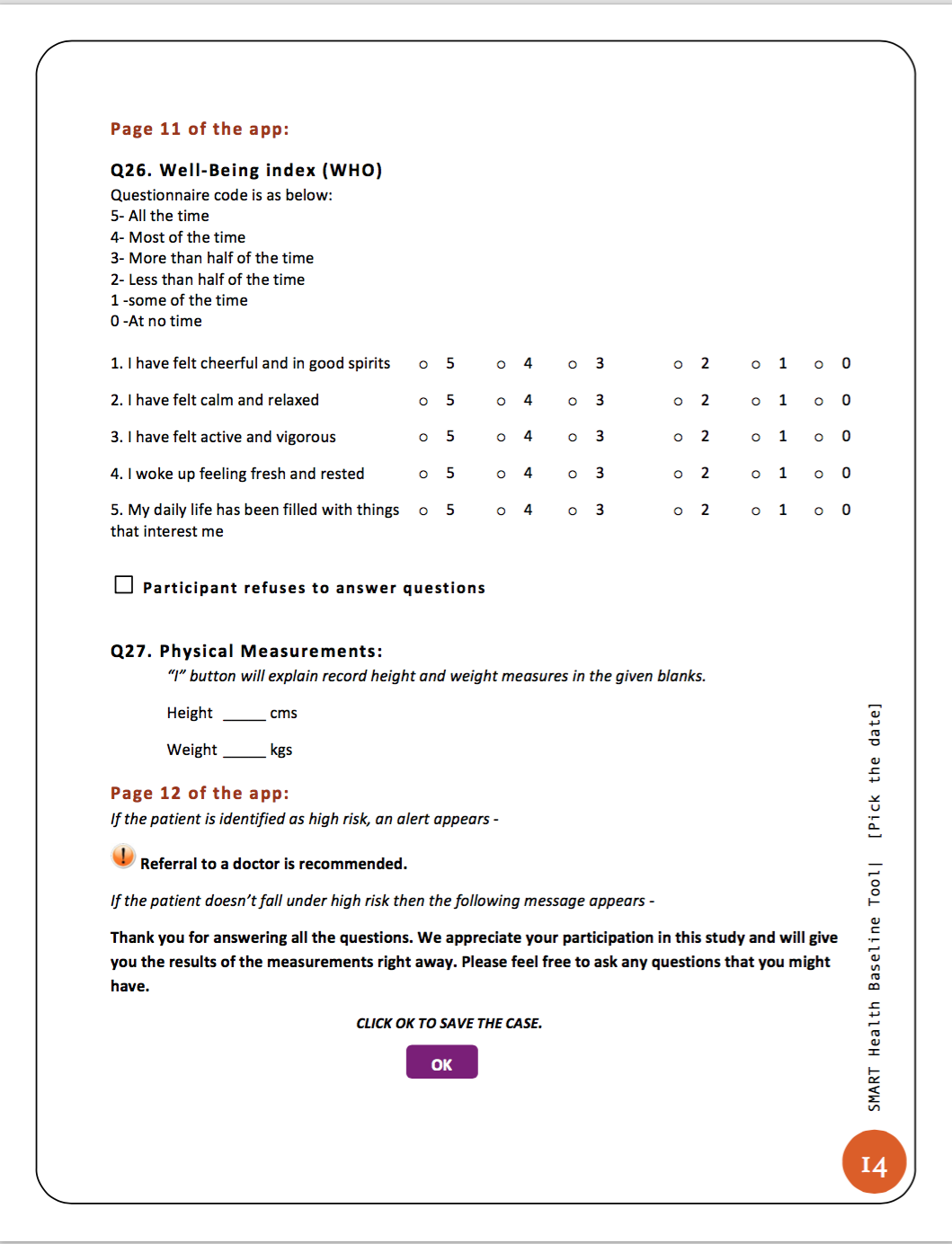

Supplement: S1 Appendix — (DOCX) [file pone.0215219.s002.docx]
